# Morphine Induces Expression of Platelet-Derived Growth Factor in Human Brain Microvascular Endothelial Cells: Implication for Vascular Permeability

**DOI:** 10.1371/journal.pone.0021707

**Published:** 2011-06-28

**Authors:** Hongxiu Wen, Yaman Lu, Honghong Yao, Shilpa Buch

**Affiliations:** Department of Pharmacology and Experimental Neuroscience, University of Nebraska Medical Center, Omaha, Nebraska, United States of America; Boston University School of Medicine, United States of America

## Abstract

Despite the advent of antiretroviral therapy, complications of HIV-1 infection with concurrent drug abuse are an emerging problem. Morphine, often abused by HIV-infected patients, is known to accelerate neuroinflammation associated with HIV-1 infection. Detailed molecular mechanisms of morphine action however, remain poorly understood. Platelet-derived growth factor (PDGF) has been implicated in a number of pathological conditions, primarily due to its potent mitogenic and permeability effects. Whether morphine exposure results in enhanced vascular permeability in brain endothelial cells, likely via induction of PDGF, remains to be established. In the present study, we demonstrated morphine-mediated induction of PDGF-BB in human brain microvascular endothelial cells, an effect that was abrogated by the opioid receptor antagonist-naltrexone. Pharmacological blockade (cell signaling) and loss-of-function (Egr-1) approaches demonstrated the role of mitogen-activated protein kinases (MAPKs), PI3K/Akt and the downstream transcription factor Egr-1 respectively, in morphine-mediated induction of PDGF-BB. Functional significance of increased PDGF-BB manifested as increased breach of the endothelial barrier as evidenced by decreased expression of the tight junction protein ZO-1 in an *in vitro* model system. Understanding the regulation of PDGF expression may provide insights into the development of potential therapeutic targets for intervention of morphine-mediated neuroinflammation.

## Introduction

Opiates abuse has been implicated as a contributing risk factor for increased neuroinflammation associated with various neurodegenerative disorders. Increasing often used for pain management has been shown to not only accelerate HIV-1 replication while causing immunosuppression, but can also exacerbate neuropathogenesis associated with AIDS [1], [2], [3], [4]. In fact, evidence of more severe pathology has been observed at autopsy [5] in patients with cancer metastasis, kidney and lung disease and in brains of HIV-1 infected individuals that were opiate users. Despite the recognized impact of the abuse of morphine on the disease pathology, the underlying mechanisms involved in this process remain elusive.

In our previous study we have demonstrated increased expression of PDGF in the brains of macaques with SIV-encephalitis [6]. Specifically, PDGF-BB was present in perivascular macrophages lining the small blood vessels in the brain [6]. In this previous study, however, the contribution of endothelial cells as a source of PDGF-BB was not investigated. The present study was undertaken to examine whether morphine exposure of human brain microvascular endothelial cells (HBMECs) resulted in increased PDGF-BB expression, and if so, did this overexpresssion lead to disruption of endothelial cell integrity.

PDGF is a known mitogen & chemoattractant for a number of cell types both *in vitro* and *in vivo* [7], [8] and can activate transcription of a number of otherwise quiescent genes, several of which are known to encode potent cytokines and proto-oncogenes [9]. To date four known PDGF ligands (A–D) have been identified that can exert their effect via specific cell membrane tyrosine kinase receptors designated α & β [10], [11]. PDGF has been implicated in pathologies of the lung, heart, brain and various tumors [6], [12]. Its role in drug abuse-induced CNS complication however, remains elusive. Recent intriguing findings by Su et al have implicated PDGF released from astrocytes as a critical factor in the disruption of cerebrovascular permeability leading to increased risk for stroke [12].

Blood-brain barrier (BBB) is critical for the maintenance of CNS homeostasis and for the regulation of the neuronal microenvironment. While neuroinflammation is one of the hallmark features of morphine-induced CNS aberration [1], information on factors mediating the BBB breach is scanty. Based on its role as a vascular permeant, we hypothesized that PDGF-BB was critical for inducing morphine-mediated disruption of the endothelial barrier.

The present study was aimed at exploring the signaling mechanisms by which morphine mediated induction of PDGF-B chain in HBMECs and its functional implications for increased neuroinflammation. Specifically, this study aims to focus on the mitogen-activated protein kinase mediators such as Erk, JNK,p38 and PI3K/Akt pathways that have previously been shown to be regulated by morphine [13], [14]. Understanding the regulation of PDGF expression by morphine may provide insights into the development of potential therapeutic targets aimed at intervening influx of inflammatory cells into the CNS.

## Materials and Methods

### Materials

Morphine and the opioid receptor antagonist naltrexone were purchased from Sigma (St. Louis, MO). The specific Phosphatidinylinositol-3′ (PI3K) inhibitor LY294002, MEK inhibitor U0126, JNK inhibitor SP600125 and P38 inhibitor SB203580 were obtained from Calbiochem (San Diego, CA). Chromatin immunoprecipitation (ChIP) assay kit was purchased from Upstate (Billerica, MA). Dominant negative and constitutively active constructs of Egr-1 were provided by Dr. Young Han Lee (Konkuk University, Korea).

### Cell culture and treatment

Primary HBMECs obtained from Dr. Monique Stins (Johns Hopkins University) were cultured in RPMI 1640 medium containing 10% heat-inactivated fetal bovine serum, 10% Nu-Serum, 2 mM glutamine, 1 mM pyruvate, penicillin (100 units/ml), streptomycin (100 µg/ml), essential amino acids, and vitamins. Purified HBMECs were positive for endothelial makers DiI-AcLDL (left panel), ZO-1 (middle panel) and β-catenin (right panel) and were found to be >99% pure after exclusion of staining for non-endothelial cell type markers (GFAP, smooth muscle actin, cytokeratin and macrophage antigens) as described previously [15]. For pharmacological inhibition studies, cells were pre-treated for 1 h with naltrexone (10 µM), Phosphatidinylinositol-3′ (PI3K) inhibitor LY294002 (10 µM), MEK inhibitor U0126 (20 µM), JNK inhibitor SP600125 (20 µM), and P38 inhibitor SB203580 (20 µM), prior to morphine treatment. The concentrations of morphine and these inhibitors were based on the dose-curve study previously reported by us [16], [17], [18]. All experiments were conducted under serum-free conditions because serum is known to induce PDGF [19].

### Real-Time PCR

Total RNA was extracted using Trizol reagent according to the manufacturer's protocol (Invitrogen Life Technologies, CA). 1 µg of RNA was used for cDNA production according to manufacturer's instructions (Thermo Scientific, MA). Human PDGF-B primer was obtained from SA Biosciences (Frederick, MD). Quantitative Analyses of mRNA were conducted using ABI 7500 Fast Real-Time PCR system (Applied Biosystems, CA). Data were normalized using Ct values for β-actin in each sample. To calculate relative amounts mRNA, the average Ct values were subtracted from β-actin values for each target gene to provide changes in Ct value. Fold Change in expression was calculated as log_2_ relative units.

### Western Blotting

Treated cells were washed with PBS buffer followed by lysis using the Mammalian Cell Lysis kit (Sigma, St. Louis, MO). Cell lysates were subjected to protein separation using 10% SDS-PAGE electrophoresis (equal amounts of the corresponding proteins, about 25 µg protein per well) followed by transferring of protein onto polyvinylidene difluoride membrane. The blots were blocked with 5% non-fat dry milk in PBS. Western blots were then probed with antibodies recognizing Egr-1 (Santa Cruz, CA, 1∶500), PDGF-BB (Santa Cruz, CA 1∶ 1000), ZO-1 (Invitrogen, CA, 1∶200), β-actin (Santa Cruz, CA, 1∶5000) and phosphorylated forms of Erk1/2, JNK, p38 and Akt (Cell Signaling, Danvers, MA, 1∶200). Signals were detected by chemiluminescence (Pierce, Rockford, IL).

### Immunocytochemistry and Imaging

Immunocytochemical analysis of Egr-1 and PDGF-BB expression were performed in morphine-treated and untreated HBMECs grown on round glass coverslips. Following morphine treatment, cells were treated with 4% paraformaldehyde for 15 min at room temperature and permeabilized with PBS containing 0.3% Triton X-100 for 30 min at room temperature. After blocking with PBS containing 10% normal goat serum, cells were incubated at 4°C overnight with either anti-Egr-1 or anti-PDGF-BB (both at 1∶200 dilution) rabbit polyclonal antibodies (Santa Cruz Biotechnology). After washing, cells were incubated with the secondary goat anti-rabbit AlexaFluor 488 and 594-conjugated antibody (1∶500) for PDGF-BB and Egr-1 respectively. For negative controls, cells were treated as described above without the primary antibody. Cells were mounted with Prolong Gold containing 4′, 6′-diamidino-2-phenylindole to stain nuclei (Molecular Probe, Eugene,OR). Immunostaining was observed using a microscope (ZEISS).

### Transfection with plasmid constructs

HBMECs were transfected with plasmid vectors containing either wild type (WT) or dominant negative (DN) forms of Egr-1 as described previously [20]. Knock-down efficiencies were determined by Western Blotting.

### Chromatin immunoprecipitation (ChIP) Assay

ChIP assay was performed according to the manufacturer's instructions. After treatment the cells with morphine, 18.5% fresh formaldehyde was added directly into the medium at a final concentration of 1% formaldehyde and incubated for 10 min at room temperature, followed by quenching with 10×glycine. The cells were then scraped using 2 ml pre-chilled PBS containing 1×protease inhibitor cocktail. The cell pellet was harvested by spinning at 800×g at 4°C followed by adding lysis buffer (provided in the kit) to harvest nuclei. DNA was sheared by sonication. 50 µl of the sheared cross-linked chromatin was then mixed with 20 µl of protein A magnetic beads and 5 µg of the corresponding antibody diluted in 450 µl dilution buffer followed by incubation overnight at 4°C. The magnetic beads binding antibody/chromatin complex was then washed with 0.5 ml each of a series of cold wash buffers in the order of low salt buffer, high salt buffer, LiCl buffer and finally with Tris-EDTA buffer. The cross-linked protein/DNA complexes were reversed to free DNA by incubation at 62°C for 2 h, and purified using DNA purification spin columns following the manufacturer's instructions. Finally the purified DNA was amplified via PCR to identify promoter region containing Egr-1 binding site “GCG GGG GCG”. The sequence of the primers used to identify the PDGF-B promoter bound to Egr-1 were as follows: sense, 5′-GCAGAGGCCTGAGCGCCTGATC-3′, anti–sense, 5′-GCAGCGATTCATGCCGACTCCG-3′.

### Cell Permeability

Primary HBMECs at passage 18 were seeded (4×10^4^cells/well) onto 6.5-mm polyester Transwell inserts (0.4-µm pore size) and grown for 5 days to achieve confluence. HBMEC monolayers were generated and treated with 10^−7^ M of morphine, in the presence or absence of naltrexone, neutralizing PDGF-BB antibody or isotype antibody for the times indicated. To detect changes in monolayer permeability, following incubation for 24 h, FITC Dextran-4 (1 mg/ml; Sigma) was added to the upper chamber of the inserts and incubation continued for additional 2 h. Aliquots (100 µl) were then collected from the lower chamber for fluorescent measurement using 480 and 530 nm for excitation and emission, respectively (Biotek Synergy MX multimode microplate reader instrument). The fluorescence intensity of each well was subtracted by that of medium alone without Transwell insert incubation. Permeability changes were expressed as percentage of FITC-dextran transported across the BBB into the lower chamber compared to untreated control cultures.

### Statistical Analysis

Statistical analysis was performed using one-way analysis of variance with a post hoc student t test. Results were judged statistically significant if p<0.05 by analysis of variance.

## Results

### Morphine mediated up-regulation of PDGF-B expression in HBMECs

**Figure 1:**
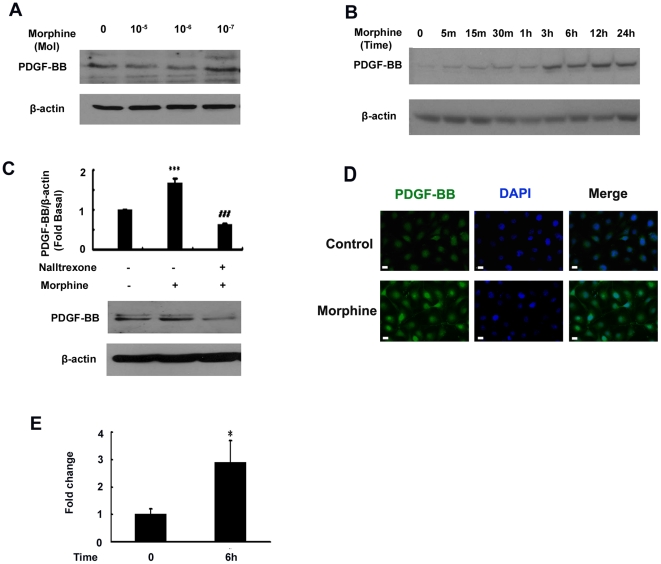
Morphine-mediated induction of PDGF-B chain expression in human brain microvascular endothelial cells. (**A**) Dose-dependent induction of PDGF-BB protein by morphine (10^−5^, 10^−6^ and 10^−7^ M) in human brain microvascular endothelial cells (HBMECs). (**B**) Time-dependent induction of PDGF-BB protein by morphine (10^−7^ M) in HBMECs. (**C**) Opioid receptor antagonist naltrexone (1 µM) abrogates morphine-mediated induction of PDGF-BB. (**D**) Representative picture of PDGF-B chain staining in HBMECs treated with morphine for 12 hrs. Scale bar = 5 µm. (**E**) Induction of PDGF-B mRNA in HBMECs exposed to morphine after 6 hrs. All the data are presented as mean±SD of three individual experiments. *p<0.05, ***p<0.001 vs control group; ###p<0.001 vs morphine group.

To assess the dose-curve of morphine effect, serum-starved HBMECs were first treated with varying concentrations of morphine (10^−5^, 10^−6^ and 10^−7^ M) for 12 h and assessed for expression of PDGF-BB by western blotting. As shown in Fig. 1A, maximal induction of PDGF-BB was at a morphine concentration of 10^−7^ M. The rationale for using this concentration range for morphine was based on our previously published report [16]. This concentration was thus used for all further experiments. The next step was to determine the time course of morphine-mediated induction of PDGF-BB. As shown in Fig. 1B, exposure of serum-starved HBMECs to morphine for varying times (5–30 min; 1–24 h) resulted in time-dependent induction of PDGF-BB with a maximal response at 12 h, followed by a gradual decline thereafter. To examine whether morphine-mediated induction of PDGF-BB involved the opioid receptor cells were also pretreated the receptor antagonist, naltrexone (1 µM) followed by treatment with morphine at 12 h. As shown in Fig. 1C, naltrexone abrogated morphine-mediated up-regulation of PDGF-BB. Confirmation of these findings by immunostaining also revealed increased expression of PDGF-BB in morphine-exposed HBMECs 12 h post-treatment (Fig. 1D). Consistent with the protein levels, morphine exposure also increased mRNA for PDGF-B chain as determined by Real Time-PCR with a maximal up-regulation (2.9 fold) at 6 h (Fig. 1E), preceding the peak protein expression at 12 h. Cumulatively, these data clearly demonstrate that exposure of HBMECs to morphine resulted in opioid receptor -mediated induction of PDGF-B chain RNA and PDGF-BB protein.

### Morphine-mediated induction of PDGF-BB via the opioid receptor involves activation of Erk1/2, JNK and p38 MAPK but not PI3K/Akt signaling pathways

**Figure 2:**
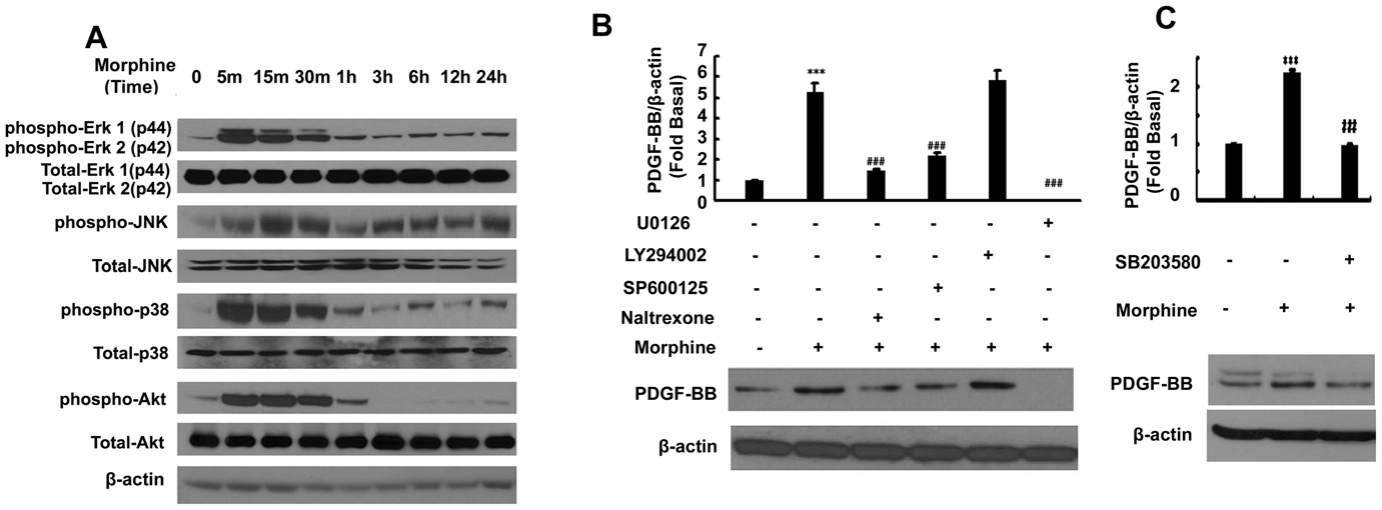
Opioid receptor, Erk1/2 and JNK MAPK but not PI3K/Akt pathways are involved in morphine-mediated induction of PDGF-BB in HBMECs. (**A**) Time-dependent activation of Erk1/2, JNK, p38 and Akt by morphine as evidenced by western blot analysis. Pretreatment of HBMECs with the inhibitors specific for Erk1/2-U0126 20 µM (B), JNK-SP600125 20 µM (B) and p38-SB203580 20 µM (C) but not Akt-LY294002 10 µM) (B) pathways abrogates morphine-mediated induction of PDGF-BB. All the data are presented as mean±SD of three individual experiments. ***p<0.001 vs control group; ###p<0.001 vs morphine group.

Having determined morphine-mediated induced expression of PDGF-B chain in HBMECs, we next sought to elucidate the signaling pathways involved in this process. Since morphine can activate mitogen-activated protein kinase (MAPK) pathways [21], we examined the involvement of Erk1/2, JNK and p38 kinases in morphine-induced expression of PDGF-BB. Treatment of HBMECs with morphine resulted in a time-dependent increase in phosphorylation of Erk1/2, JNK, p38 and Akt (Fig. 2A).

Next, we wanted to know whether morphine-mediated induction of PDGF-BB involved activation of these signaling pathways. HBMECs were pre-treated with inhibitors specific for the respective signaling pathways prior to stimulation with morphine and assessed for expression of PDGF-BB. As shown in Fig. 2B–C, pre-treatment of cells with inhibitors specific for MEK (U0126, 20 µM), JNK (SP600125, 20 µM), p38 (SB203580, 20 µM) but not PI3K (LY294002, 10 µM) resulted in amelioration of morphine-mediated induction of PDGF-BB. These findings underpin the role of Erk1/2, JNK and p38 MAPKs but not PI3K/Akt cascade in morphine-mediated induction of PDGF-BB in HBMECs.

### Transcription factor Egr-1 is activated in HBMECs exposed to morphine

**Figure 3:**
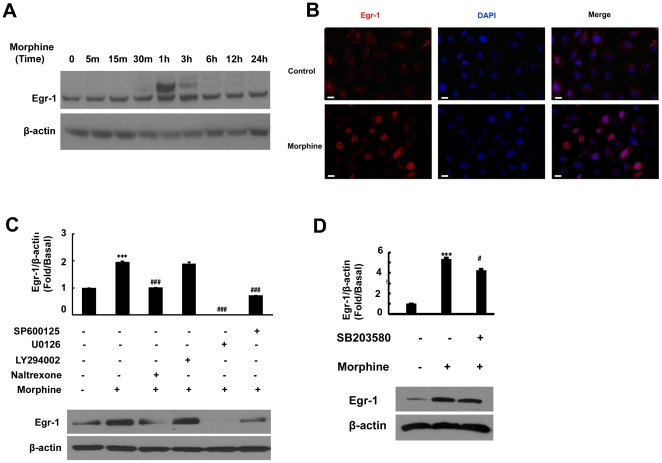
Egr-1 expression is up-regulated in HBMECs exposed to morphine. (**A**) Time-dependent activation of Egr-1 protein expression by morphine in HBMECs. (**B**) Representative image of Egr-1 staining in HBMECs treated with morphine. Scale bar = 5 µm. Pretreatment of HBMECs with the inhibitors specific for Erk1/2-U0126 20 µM,JNK-SP600125 20 µM (**C**) and p38-SB203580 20 µM (**D**) but not Akt-LY294002 10 µM (**C**) pathways abrogates morphine-mediated induction of Egr-1. All the data are presented as mean±SD of three individual experiments. ***p<0.001 vs control group; #p<0.05, ###p<0.001 vs morphine group.

Having determined the involvement of Erk1/2, JNK and p38 MAPK in morphine-mediated induction of PDGF-BB expression, we next wanted to assess the downstream signaling mediators involved in this process. Based on the published reports demonstrating the presence of binding sequences for the transcription factor, early growth response (Egr)-1 on the PDGF-B promoter [22], we rationalized that morphine-mediated induction of PDGF-BB could likely involve the downstream effector Egr-1. Exposure of HBMECs to morphine resulted in a time-dependent increase in Egr-1 expression (Fig. 3A), with a peak expression at 1 h post-treatment and a decline thereafter. These findings were also validated by Egr-1 immunostaining demonstrating increased Egr-1 expression in morphine-treated HBMECs at 1 h post-treatment (Fig. 3B).

Next logical step was to examine whether there existed a link that could tie morphine-mediated activation of Erk1/2, JNK, p38 MAPKs and PI3K-Akt pathways with Egr-1 and, if so, the involvement of opioid receptor in this process. HBMECs were pre-treated with naltrexone, and MEK, JNK, p38 or PI3K inhibitors followed by treatment with morphine and assessed for activation of Egr-1. As shown in Fig. 3C–D, exposure of HBMECs to opioid receptor antagonist and either MEK, JNK or p38 inhibitors resulted in abrogation of morphine-mediated activation of Egr-1. Intriguingly, pre-treatment of cells with the PI3K inhibitor had no effect on morphine-mediated activation of Egr-1. These findings thus linked morphine-mediated activation of Erk1/2, JNK and p38 signaling pathways to downstream activation of Egr-1 with an involvement of opioid receptor in this process.

### Involvement of Egr-1 in morphine-induced expression of PDGF-BB in HBMECs

**Figure 4:**
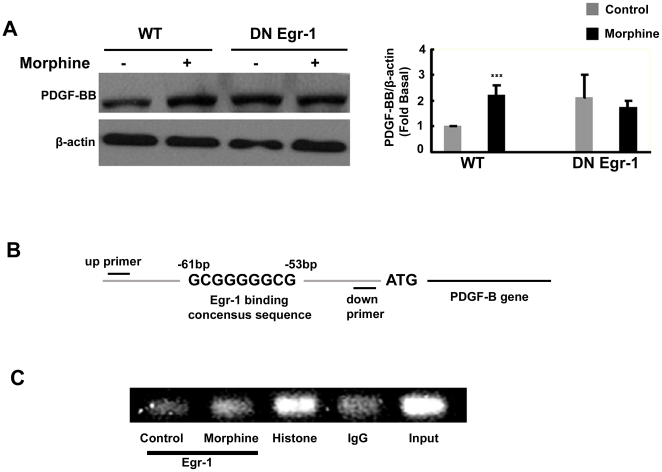
Involvement of Egr-1 in morphine-mediated induction of PDGF-BB in HBMECs. (**A**) Whole cell lysates of HBMECs transfected with either the wild type (WT) or dominant negative (DN) construct of Egr-1 were subject to western blot analysis using antibodies specific for PDGF-BB. Transfection of cells with the DN-Egr-1, but not the WT-Egr-1 inhibited morphine-mediated induction of PDGF-BB expression. (**B**) Schematic illustration of Egr-1 binding consensus sequence on the PDGF-B promoter region. (**C**) ChIP assay demonstrating morphine-mediated binding of Egr-1 to the PDGF-B promoter. All the data are presented as mean±SD of three individual experiments. ***p<0.001 vs control group.

Next we wanted to confirm the role of Egr-1 in morphine-mediated induction of PDGF-BB. HBMECs were transfected with either the WT or DN construct of Egr-1, followed by treatment with morphine and assessed for expression of PDGF-BB by WB. As shown in Fig. 4A, morphine-mediated induction of PDGF-BB was attenuated by the DN-Egr-1, but not the WT-Egr-1 construct further validating the pharmacological approach described above.

To confirm the binding of Egr-1 with PDGF-B promoter in its natural chromatin context, we performed chromatin immunoprecipitation assay to reveal active sites on the PDGF-B promoter that are accessible to Egr-1. HBMECs were treated with morphine for 1 h followed by analysis using a ChIP kit. These experiments revealed increased binding of Egr-1 to the PDGF-B promoter in HBMECs exposed to morphine (Fig. 4B–C). Collectively, these findings emphasize the role of Egr-1 in morphine-mediated induction of PDGF-BB.

### Morphine-mediated disruption of ZO-1 expression involves PDGF-BB

**Figure 5:**
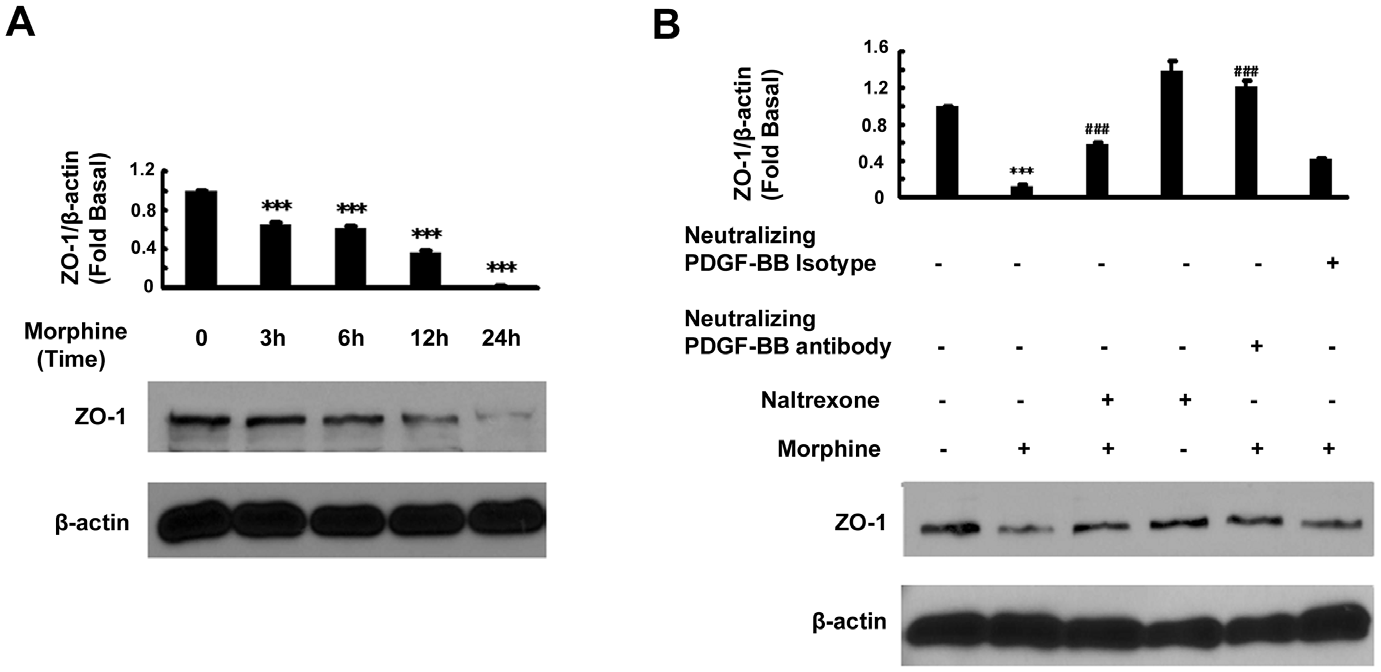
PDGF-BB induces down regulation of ZO-1 in HBMECs. (**A**) Time-dependent reduction of ZO-1 protein expression by morphine in HBMECs. (**B**) Treatment of cells with either the opioid receptor antagonist (naltrexone) or PDGF-BB neutralizing antibody results in inhibition of ZO-1 expression. All the data are presented as mean±SD of at least three individual experiments. ***p<0.001 vs control group; ###p<0.001 vs morphine-treated group.

The next step was to identify the role of up-regulated PDGF-BB in HBMECs. Since PDGF-BB is a known vascular permeant, we rationalized that morphine-mediated upregulation of PDGF-BB may be critical for disruption of the endothelial barrier likely via downregulation of the tight junction protein ZO-1. HBMECs were exposed to morphine and assessed for the expression of ZO-1 by Western blot. As shown in Fig. 5A, treatment of HBMECs with morphine resulted in a time-dependent decrease in the expression of ZO-1 and, this effect was significantly ameliorated in cells pre-treated with either naltrexone or PDGF-BB neutralizing antibody (Fig. 5B). These findings thus underscored the role of PDGF-BB in morphine/opioid receptor axis-mediated down regulation of ZO-1.

### Morphine-mediated induction of PDGF-BB increases BBB permeability

**Figure 6:**
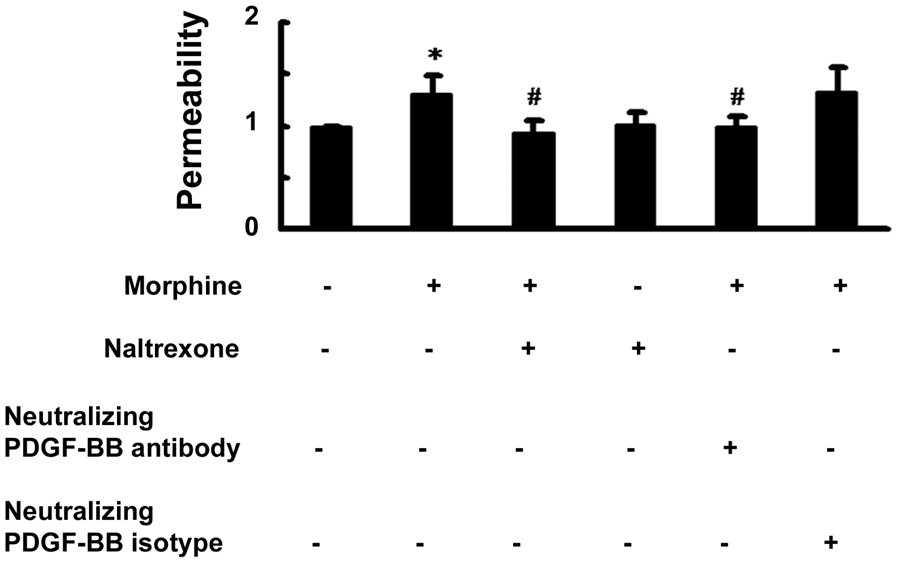
Effect of morphine on BBB permeability *in vitro*. Morphine-mediated increase in BBB permeability was ameliorated in HBMECs pretreated with either naltrexone or PDGF-BB neutralizing antibody. All the data are presented as mean±SD of four individual experiments. *p<0.05 vs control group; #p<0.05 vs morphine-treated group.

Functional relevance of PDGF-BB-mediated down-regulation of ZO-1 was further confirmed using *in vitro* permeability assays. As shown in Fig. 6, exposure of HBMECs monolayer to morphine increased monolayer permeability by almost 30%. Intriguingly, pre-treatment of HBMECs with either naltrexone or PDGF-BB neutralizing antibody significantly ameliorated morphine-induced leakiness of the BBB, thereby underpinning the role of opioid receptor and PDGF-BB in this process.

## Discussion

Mounting evidence indicated that drug abuse speeds up the progression to AIDS in HIV-infected individuals and exacerbates neurological complications associated with the disease [2], [16], [23]. Morphine increases the activation of astrocytes and macrophages/microglia in the brain through regulation of diverse pro-inflammatory cytokines and chemokines [24], [25]. Morphine has also been shown to alter BBB function by regulating the expression of pro-inflammatory cytokines (TNF-α and IL-8) and/or transmembrane protein (P-glycoprotein) [1], [26]. However, very few studies have actually explored direct effects of morphine on the expression of a cerebrovascular permeant PDGF-BB. Our study demonstrates for the first time that morphine-mediated disruption of BBB involves up-regulation PDGF-BB in HBMECs.

In the present study we demonstrated that exposure of HBMECs to morphine resulted in induction of PDGF expression at both the transcriptional and translational levels through the binding of morphine to the opioid receptor. Further dissection of the signaling pathways involved in morphine-mediated induction of PDGF-BB using pharmacological and genetic approaches revealed activation of Erk, JNK, p38 MAPKs and also PI3K/Akt pathways, a finding that is consistent previous reports on the effect of morphine in neurons [27]. Morphine-mediated induction of PDGF-BB expression however, involved Erk, JNK and p38 MAPKs, but not PI3K/Akt pathway. These findings are in agreement with previous reports demonstrating morphine-mediated activation of the MAPK and PI3K-Akt pathways, similar to the effects of vascular endothelial growth factor (VEGF) on mouse retinal endothelial cells [13], [14]. It is likely that morphine-induced activation of MAPKs and PI3K/Akt pathways could be due to the co-activation of VEGFR2 and PDGF-βR [28]. In our studies morphine increased endothelial permeability in HBMECs, which is in keeping with a pervious reciprocal finding that mu opioid receptor-specific antagonist methylnaltrexone inhibited morphine-induced vascular permeability [29]. It must be pointed out that in another unrelated paradoxical study, morphine has been suggested to inhibit expression of another vascular permant such as VEGF that is induced by hypoxia both in endothelial cells as well as cardiac myocytes [30], [31]. One possible explanation for this disparity in regulation of VEGF versus PDGF, could be attributed to the fact that since both these factors belong to a family of related growth factors, upregulation of PDGF could be a compensatory mechanism for reduced VEGF expression mediated by morphine.

The transcription factor, Egr-1, has been found to be a major regulatory transcription factor for PDGF-BB in several types of cells [32], [33]. In this study, our findings demonstrated a time-dependent up-regulation of Egr-1 protein in HBMECs (Fig. 3A), and the CHIP assay results confirmed the regulation of Egr-1 in the transcriptional activation of PDGF-BB (Fig. 4C). Further dissection of Egr-1 regulation using both the pharmacological and genetic approaches revealed the activation of Erk, JNK and p38 MAPKs pathways upstream of Egr-1 (Fig. 2). In agreement with our findings, activation of Erk, JNK Egr-1 pathway has also been shown to play a role in FGF-2 treated astrocytes [34].

To determine a link between Egr-1 and PDGF-BB expression, we also demonstrated increased Egr-1 binding to the PDGF-B promoter in morphine-treated HBMECs (Fig. 4), thus confirming the role of Egr-1 in PDGF-BB expression. Further support of Egr-1 involvement in morphine-mediated PDGF-BB induction was also demonstrated using genetic approaches. Our findings are in agreement with the report by Khachigan *et al*. demonstrating interaction of Egr-1 with the PDGF-B promoter in arterial endothelial cells [22].

Functional implication of morphine-mediated induction of PDGF-BB was also assessed in an *in vitro* model of BBB. Exposure of HBMECs to morphine resulted in down-regulation of the tight junction protein ZO-1 with the involvement of both MOR and PDGF-BB as evidenced in cells treated with the natlrexone and PDGF-B neutralizing antibody, respectively. These findings are in agreement with the recent studies implicating the role of PDGF family members as mediators of BBB damage [12], [20].

In summary, our findings demonstrate a detailed molecular pathway of morphine-mediated induction of PDGF-BB in HBMECs, involving engagement of opioid receptor and activation of Erk, JNK and p38 MAPK pathways and the downstream transcription factor Egr-1 leading to increased expression of PDGF-BB, culminating ultimately into BBB damage. These findings have implications for HIV-1-infected heroin abusers that are known to have increased risk of neuroinflammation.
